# Massively Parallel Sequencing Reveals the Complex Structure of an Irradiated Human Chromosome on a Mouse Background in the Tc1 Model of Down Syndrome

**DOI:** 10.1371/journal.pone.0060482

**Published:** 2013-04-15

**Authors:** Susan M. Gribble, Frances K. Wiseman, Stephen Clayton, Elena Prigmore, Elizabeth Langley, Fengtang Yang, Sean Maguire, Beiyuan Fu, Diana Rajan, Olivia Sheppard, Carol Scott, Heidi Hauser, Philip J. Stephens, Lucy A. Stebbings, Bee Ling Ng, Tomas Fitzgerald, Michael A. Quail, Ruby Banerjee, Kai Rothkamm, Victor L. J. Tybulewicz, Elizabeth M. C. Fisher, Nigel P. Carter

**Affiliations:** 1 Wellcome Trust Sanger Institute, Wellcome Trust Genome Campus, Hinxton, Cambridge, United Kingdom; 2 Department of Neurodegenerative Disease, UCL Institute of Neurology, Queen Square, London, United Kingdom; 3 MRC National Institute for Medical Research, The Ridgeway, Mill Hill, London, United Kingdom; 4 Health Protection Agency Centre for Radiation, Chemical & Environmental Hazards Chilton, Didcot, Oxon, United Kingdom; The Walter and Eliza Hall of Medical Research, Australia

## Abstract

Down syndrome (DS) is caused by trisomy of chromosome 21 (Hsa21) and presents a complex phenotype that arises from abnormal dosage of genes on this chromosome. However, the individual dosage-sensitive genes underlying each phenotype remain largely unknown. To help dissect genotype – phenotype correlations in this complex syndrome, the first fully transchromosomic mouse model, the Tc1 mouse, which carries a copy of human chromosome 21 was produced in 2005. The Tc1 strain is trisomic for the majority of genes that cause phenotypes associated with DS, and this freely available mouse strain has become used widely to study DS, the effects of gene dosage abnormalities, and the effect on the basic biology of cells when a mouse carries a freely segregating human chromosome. Tc1 mice were created by a process that included irradiation microcell-mediated chromosome transfer of Hsa21 into recipient mouse embryonic stem cells. Here, the combination of next generation sequencing, array-CGH and fluorescence *in situ* hybridization technologies has enabled us to identify unsuspected rearrangements of Hsa21 in this mouse model; revealing one deletion, six duplications and more than 25 *de novo* structural rearrangements. Our study is not only essential for informing functional studies of the Tc1 mouse but also (1) presents for the first time a detailed sequence analysis of the effects of gamma radiation on an entire human chromosome, which gives some mechanistic insight into the effects of radiation damage on DNA, and (2) overcomes specific technical difficulties of assaying a human chromosome on a mouse background where highly conserved sequences may confound the analysis. Sequence data generated in this study is deposited in the ENA database, Study Accession number: ERP000439.

## Introduction

Down syndrome is the most common genetic cause of intellectual disability, accounting for ∼1 in 750 births, and is caused by trisomy of chromosome 21 [1]. The syndrome consists of a complex phenotype of a few ‘invariant’ features that appear in all affected individuals, such as the cognitive abnormalities and early-onset Alzheimer’s disease pathology, and at least 80 variable features, all of which are also found to different extents in the euploid population [2]. Up to 8 million people globally are estimated to have DS, and thus this disorder has a considerable societal and clinical impact. DS also represents a fascinating molecular genetics problem – we know most of the structure of Hsa21, but we are only at the very beginning of making genotype-phenotype correlations and working out which of the genes on the chromosome are dosage sensitive and hence result in changes to phenotype when their copy number is altered.

To help model and understand the molecular genetics of DS, the first transchromosomic mouse, the Tc1 model (Tc(Hsa21)1TybEmcf) was created, which carries a freely segregating copy of human chromosome 21 [3]. This model was generated by irradiation microcell-mediated chromosome transfer (XMMCT); briefly Hsa21 was isolated in microcells from a human cell-line (HT1080) ([4]) and γ-irradiated before transfer into a 129S2 mouse embryonic stem (ES) cell line**.** ‘Transchromosomic’ ES cells were then injected into recipient blastocysts which were allowed to develop to term. Resulting chimeric animals were bred and a single germ-line transmission of an irradiated Hsa21 led to the establishment of the Tc1 mouse strain.

The Tc1 mouse strain is freely available and has been widely studied as a mouse model of various aspects of DS; for example it has deficits in learning and memory [5], [6], the haematopoietic system [7], heart defects [8], and deficits in angiogenesis that may relate to the diminished frequency of specific solid tumours reported in DS [9]. This mouse has also given insight into fundamental cellular processes – Wilson, Odom and colleagues studied how mouse transcription factors bind to human promoters on Hsa21 in Tc1 tissues [10].

Previous low-resolution genetic analysis revealed that Hsa21 in the Tc1 mouse model (Tc1-Hsa21) was not intact and that not all cells in the model carry the transchromosome [3]. Thus, to fully establish the complete genomic status of the chromosome to inform and understand functional studies of the mouse model, and to investigate the effect of irradiation on a human chromosome, we undertook a detailed analysis of Tc1-Hsa21. This lead to massively parallel sequencing of the chromosome and tackling the technical and bioinformatic difficulties that arose from analysing a human chromosome on a mouse genetic background. Our results have shown unexpected rearrangements and mutation in the chromosome, have given new insight into the effects of gamma radiation on single chromosomes and have shown how the challenge of sequencing a mammalian chromosome on another mammalian background can be overcome.

## Results

### Initial High-resolution Oligonucleotide Microarray Data

**Figure 1:**
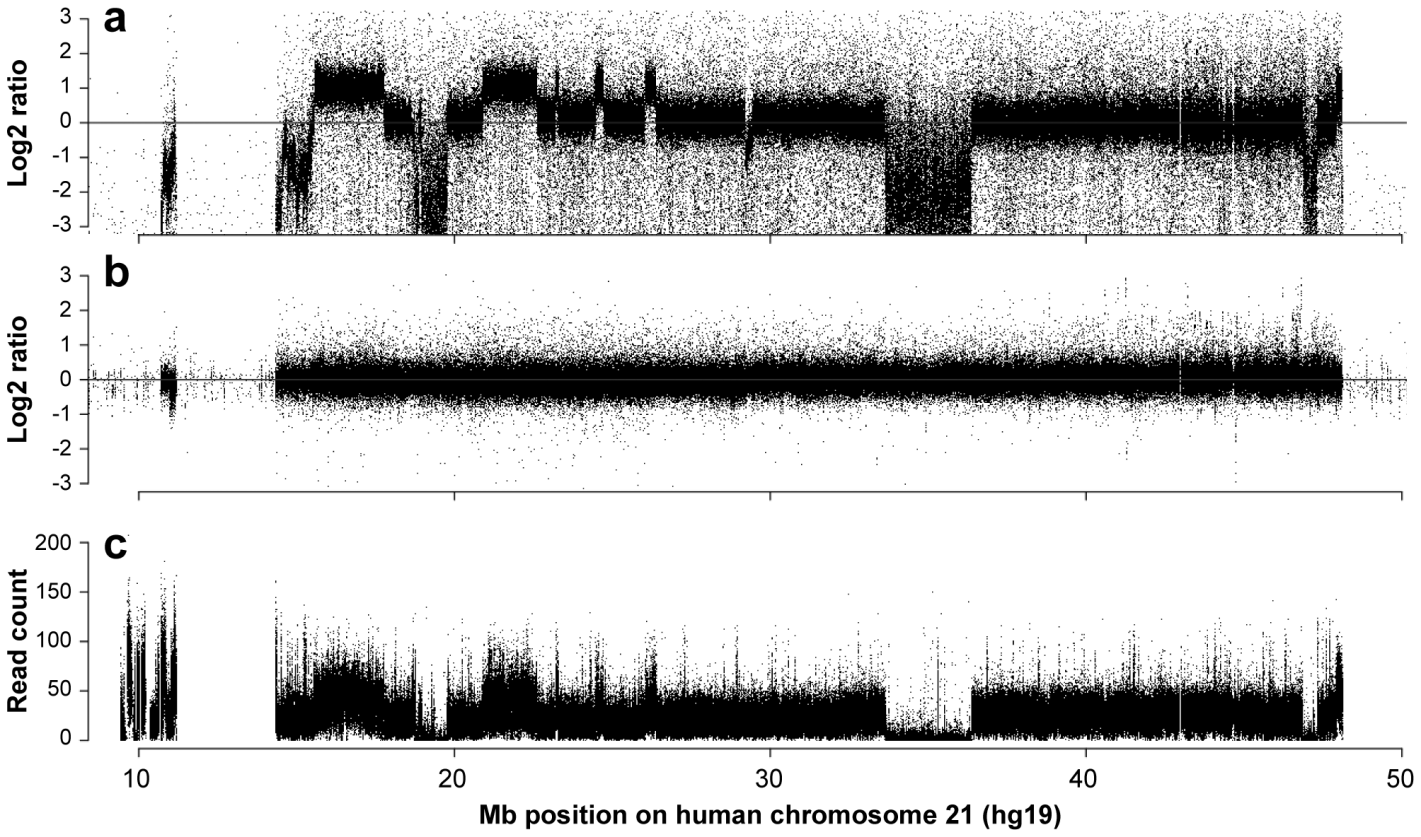
Copy number analysis of the Hsa21 in Tc1 mice. (*a*) High resolution oligonucleotide microarray comparative genomic hybridisation of Hsa21 in Tc1 mice against a human male pool reference DNA. (*b*) High resolution oligonucleotide microarray comparative genomic hybridisation of DNA extracted from the cell line HT1080 from which the Tc1 Hsa21 was originally isolated against a human male pool reference DNA. (*c*) Read depth data for Tc1 Hsa21 from NGS mapped to human chromosome 21 reference from five read paired-end sequence libraries with all the data binned at 10 bp intervals.

Initially we used a custom, high-resolution oligonucleotide microarray to obtain Tc1-Hsa21 copy number data and defined copy number change point locations (Fig. 1, Table S1). This confirmed two previously reported deletions and redefined their size i.e. delchr21∶18,734,534–19,762,829 (959 kb, note 18,873,605–18,943,066 retained) and delchr21∶33,640,510–36,370,035 (2.7 Mb). This study also revealed one novel deletion delchr21∶46,869,870–47,319,181 (449 kb) and six new duplications; dupchr21∶15,562,050–17,771,307 (2.21 Mb), dupchr21∶20,894,137–22,614,749 (1.72 Mb), dupchr21∶23,199,170–23,294,347 (95 kb), dupchr21∶24,460,290–24,726,025 (266 kb), dupchr21∶26,039,228–26,376,269 (337 kb), and dupchr21∶47,916,776–48,096,110 (179 kb) (National Center for Biotechnology Information (NCBI) build 37). Each copy number change point interval was evaluated for known copy number variation (Table S1). Parallel array Comparative Genomic Hybridisation (aCGH) of the HT1080 cell line demonstrated that Tc1-Hsa21 copy number changes were likely acquired during XMMCT or during culture selection (Fig. 1, Table S2).

### Next Generation Sequencing Analysis

To determine the genomic content of Tc1-Hsa21 further, we analysed the chromosome using next generation sequencing (NGS) technology using paired-end reads. We generated sequence data from libraries prepared from Tc1 genomic DNA or flow sorted Tc1-Hsa21 (Table S3, Fig. S1). The copy number imbalances detected by our microarray analysis were confirmed by mapping of sequence reads to the Hsa21 reference genome (NCBI37) using MAQ (Fig. 1). These data demonstrated that 8.7% of the Tc1-Hsa21 chromosome is deleted and 10.0% is duplicated. As a consequence, 45 RefSeq genes (NCBI RefSeqGene Project, http://www.ncbi.nlm.nih.gov/projects/RefSeq/RSG/, RefSeq release 42) are fully or partially deleted and nine RefSeq genes are completely duplicated. In addition, two genes (*LIPI* and *NCAM2*) are partially duplicated whilst two genes (*DIP2A* and *c21orf34*) are partially duplicated and the original copy is disrupted by further rearrangement (Table S4). We were able to confirm that 200 RefSeq genes are present in one copy in Tc1-Hsa21 and thus elucidated which genes are trisomic in this aneuploid model of Down syndrome.

**Figure 2:**
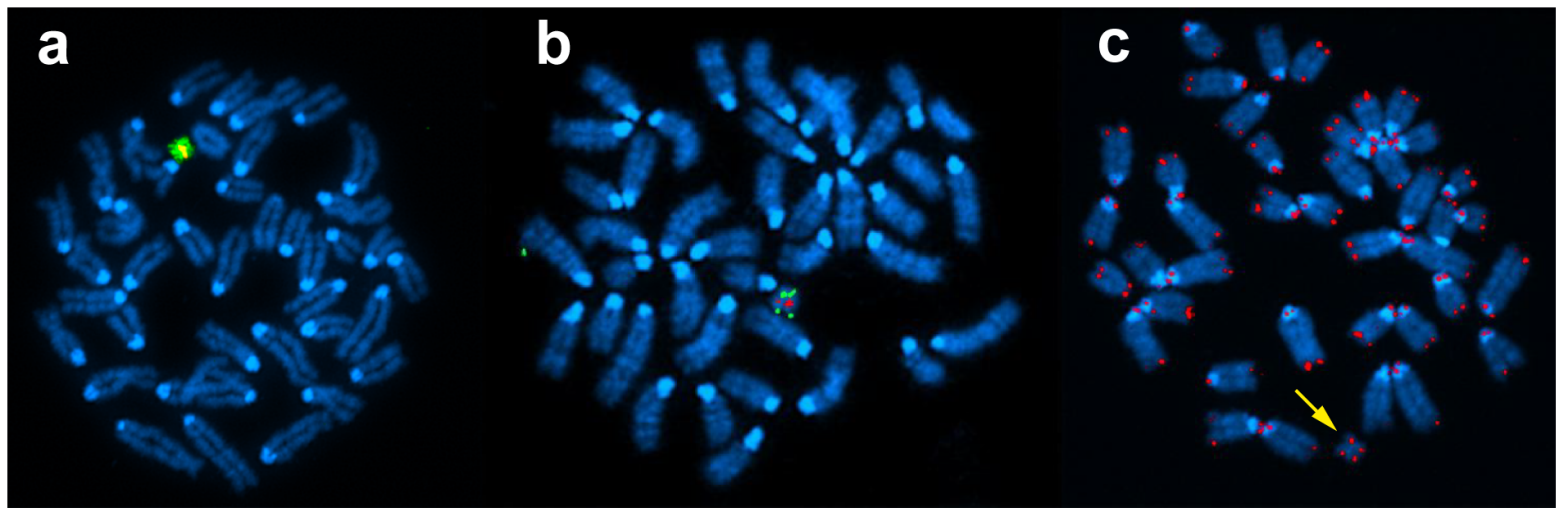
Fluorescence *in situ* hybridisation characterisation of the structure of the Hsa21 in Tc1 mice. (*a*) Hsa21 specific paint (green) c-hybridised with a Hsa13/21 alpha satellite centromere probe (red giving yellow signal). (*b*) Hsa21 telomere specific probe (green) co-hybridised with an Hsa13/21 alpha satellite centromere probe (red). (*c*) Human chromosome pan-telomeric probe (red) i.e. hybridises to all human and mouse pan telomere sequences, demonstrating that Hsa21 in the Tc1 is structurally altered and is metacentric.

Structurally, the telomeres of Tc1-Hsa21 are intact but the position of the chromosome’s centromere is altered such that the chromosome becomes metacentric (Fig. 2a–c, Fig. S2). This highlights a significant alteration of chromosomal structure which we investigated using paired-sequence reads that were found to map to more than one genomic location and hence may identify the breakpoints of the rearrangements observed (Table 1).

**Table 1:** Structural rearrangement breakpoints.

Tc1-Hsa21 break point position	Base pairs deleted at proximal breakpoint	Base pairs deleted at distal breakpoint
←9700293 **T** 37017235←▪		
←10208816 24726221→▪		
←10708238 27263491→▪		
←10603042 800 bp alpha satelite 47916774→•		
←10739714 **TG** 26376257←		
→11085336 18942901←▪		18,943,090–19,761,690
←11086535 24460837→▪•		
→11167165 25728012←		25,728,011–25,728,022
→15970237 16083578→	15,970,237–15,970,259	
←17769030 26038660→		26,038,424–26,038,659
→17764356 **GG** 17771310←		
←17770361 **C** 19761690→•		18,943,090–19,761,690
→18734159 **ACTCCTGAAATCCCAACACTTTGGGAGGC** 24880958→• ††	18,734,399–18,873,580	24,880,850–∼24,880,910
→20514026 **CTT** 24460819←		
←20514023 20756929→ ▴		20,756,923–20,756,928
→20756922 **AG** 23648470→ ▴	20,756,923–20,756,928	
→20893994 **C** 23648476←		
AT←20893996 TATATTATATATTATATATTATATA24725875←		24,725,876–24,726,137
→22612235 950bp Chr4 * 23294101←•		
←23198373 ATATAAATATA23306917→ †		
→23306930 **T** 25728022→ †		25,728,011–25,728,022
→26038424 **T** 27263316←	26,038,424–26,038,659	
→ 33223436 **CC** 36370289→	33,223,437–33,223,572	33,640,333–36,370,288
←33223573 41bp Un 49bp Chr21 ** 55bp Un 45795618→	33,223,437–33,223,572	
←37017239 45795619←		
AT→44314438 TAATATATAATATATATATTAATATATATATATATATTATATAT 47322101→♦	44,314,439–∼44,314,673	
→46868313 CATATC 47916772←	46,868,314–47,322,100	

Base pair resolution breakpoint obtained by sequencing a PCR amplified junction fragment; deletion details any loss of sequence associated with the breakpoint;

▪indicates breakpoint was not PCR verified so position is not accurate to base pair resolution;

•indicates the rearrangement was only detected in paired-sequence read data obtained in large insert libraries,

†† indicates breakpoint is between 24880910 and 24880958,

* indicates insertion that’s only known homology is with an un-mapped region on human chromosome 4 (chr4_gl000194_random, 90017–190455), Un indicates bases of unknown origin,

** indicates chr21 9826532–9826580,

▴indicates bases 23648470–23648476 appear in 2 breakpoint junction fragments,

† indicates bases 23306917–23306930 appear in 2 breakpoint junction fragments,

♦indicates AT repeat at the breakpoint is most likely to come from the 44314438 side of the breakpoint. Underlined bases are inserted at the breakpoint. Bold bases could originate from either reference sequence.

### Confirming Rearrangement Data

**Figure 3:**
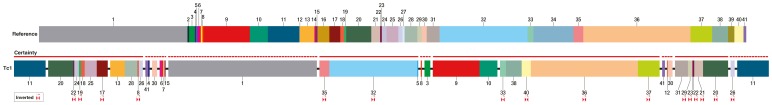
Schematic of the proposed structure of Tc1-Hsa21. Reference: Ideogram of human chromosome 21, numbers 1–41 indicate regions of Tc1 Hsa21 delineated according to Table S5. Tc1: rearranged structure of the Hsa21 in Tc1 mice. The order of regions 11, 20, 22, 24, 19, 18, 25, 17, 13, 28, 26, 6, 15, 35, 32, 5, 9, 10, 33, 38, 40, 36, 37, 31, 29, 23, 22, 21, 20, and 11 in this schematic are based on FISH mapping data (Fig. S2). The certainty of the rearrangement is indicated by a red line, solid line more certain, dotted line suggested. Inverted chromosome regions are indicated by the red arrow symbol. Region 12 is triplicated but the position of the other two copies is unknown. Position of region 27 is unknown. The positions of acrocentric regions 1, 2, 3, 7 and 8 are unknown and are placed arbitrarily. Regions 26, 30 and 41 are duplications and their positions are suggested by FISH within the resolution of the technique. Regions 14, 16, 34 and 39 are deleted.

Rearrangements seen at least twice, and in three or more sequence libraries were confirmed by breakpoint junction PCR (Table 1, Fig. S3, S4). We used a combination of chromosome breakpoints (identified from sequencing copy number changes) and aberrantly mapping read pair data (sequence reads that map to two separate genomic regions) to define genomic regions of Hsa21 that are intact in the Tc1 model. These data segmented Tc1-Hsa21 into 41 regions (Table S5). Aberrantly mapping read pair data joined regions together and mapping of these sections by FISH helped to elucidate the genomic structure of Tc1-Hsa21 (Fig. 3, Fig. S2, Table S5). We were able to show that each duplicated region was located on different arms of the rearranged Tc1 mouse Hsa21, (Fig. S2 k–p). Hybridization of a probe against the *TPTE* gene (Fig. 2, Table S5, S6), which is located on the short arm of Hsa21 close to the centromere, showed that in Tc1-Hsa21, *TPTE* is moved to near the middle of the short arm supporting the proposed rearrangement of short arm sequences (Fig. S2 g–h). The consequences of copy number changes and structural rearrangements on gene status in Tc1 Hsa21 are summarised in Table S4.

### Disruption of Specific Genes

The above rearrangements are predicted to disrupt 11 genes (Table S4). For example, the final coding exon of the amyloid beta (A4) precursor protein gene (*APP*), implicated in Alzheimer’s disease, is repositioned by a rearrangement and relocated to the other Tc1 Hsa21 chromosome arm in relation to the rest of the gene. As a consequence, this exon is not expressed in the transcript of this gene such that *APP* is not functionally trisomic in the Tc1 mouse ([3] and data not shown). Consistent with this rearrangement, expression of full-length APP transcript cannot be detected in the Tc1 brain [11] and no human APP protein is found in the Tc1 brain (personal communication, Paul Mathews).

We specifically searched for potential fusion genes; three of the genomic rearrangements disrupted a gene on both sides of the breakpoint but only one rearrangement formed an in frame coding fusion gene (Tables S7). This rearrangement may result in the generation of a potential new gene composed of the first exon of *NDUFV3* and the final 9 exons of *PCBP3*. RT-PCR has confirmed the transcription of this fusion product in Tc1 mice (Figure S5). If translated this gene would result in a novel protein composed of the first 15 amino acids of *NDUFV3*’s mitochondrial targeting sequence and the C-terminal 258 amino acids of *PCBP3*, which contain 2 RNA binding KH domains. *PCBP3* has been suggested to have a role in RNA splicing, notably a previous study has shown that these 2 C-terminal KH domains are insufficient for the protein’s role in the splicing of tau exon 10 [12], thus the hypothetical novel fusion protein may not be able to splice RNA. The MitoProt program predicts that this putative protein would be targeted to the mitochondria and hence potentially could be imported into this compartment (http://ihg2.helmholtz-muenchen.de/ihg/mitoprot.html) [13]. Regulation of mitochondrial transcripts differs significantly from nuclear/cytoplasmic RNA regulatory process, thus the functional impact of the potential novel protein is impossible to predict.

### Changes Induced by Gamma Irradiation

The 41 gross rearrangement breakpoints present in the 200 Gy-irradiated Tc1-Hsa21 chromosome were frequently associated with 1–4 bp micro-homologies but not with large regions of homologous sequence (Table 1, Fig. S3). Furthermore large-scale duplications that were unique to the irradiated Tc1-Hsa21 and not found in chromosome 21 in the HT1080 cell-line were mostly situated in Giemsa dark regions. In addition to the large scale genomic rearrangements observed in Tc1-Hsa21 46,383 SNPs relative to the reference sequence were identified; 257 of the 46,383 SNPs were categorised as stop­-gained, splice-site, nonsynonymous, or synonymous and 32 of these had not been previously reported in dbSNP (Table S8). Five of the 32 changes were detected in >95% of sequence reads (consistent with only one copy of Hsa21, as would be expected in this model). The frequency of SNPs observed in Tc1-Hsa21 is consistent with the low level of point mutations (on the order of 10) expected, assuming a gene mutation frequency of 1×10^−6^ per Gy and gene target size of 1 kbp [14].

The five microRNAs (mirbase.org Release 14) on human chromosome 21 are all present at copy number 1, are devoid of SNP mutations and not affected by the structural rearrangements present in Tc1-Hsa21.

## Discussion

We have taken a massively parallel approach using next generation sequencing to determine the DNA sequence structure of the human chromosome in the first transchromosomic mouse line to model a human disorder, Down syndrome. This is a disorder of aneuploidy, abnormal chromosome dosage and thus abnormal gene dosage. Here we had a unique opportunity to assess the DNA sequence of a human chromosome on a euploid mouse background and, further, to assess the effects of gamma radiation on the sequence of an entire human chromosome. We found unexpected changes due to irradiation providing new insight into the mechanisms of DNA damage from gamma radiation.

### Using FISH to Clarify NGS Data

Of particular note, we found we needed to employ classical FISH experiments to supplement NGS to determine the structure of the Tc1-Hsa21, as the breakpoints of some rearrangements were not covered by aberrant mapping read pairs despite the sequencing coverage obtained. Aberrantly mapping read pairs that map to sequence on the short arm of Hsa21 (Table 1) are difficult to position precisely and to confirm by PCR because of the repetitive nature of the Hsa21 p arm. This highlights a technical difficulty of NGS as some key genomic sequences, particularly those in repetitive regions prone to rearrangement, are less well determined using current platforms.

### Sequencing a Human Chromosome on a Mouse Background

Due to the unusual chromosome complement in the Tc1 mouse model we adopted two approaches to sequence the Hsa21. Initially we separated the Hsa21 away from the mouse chromosomes using flow cytometry; the smaller size of the Hsa21 compared to the mouse chromosomes allowed it to be isolated and used as an enriched source of input Hsa21 DNA for sequencing libraries (Fig. S1, Table S3). The effect of this procedure is to achieve a higher sequence read depth of the Hsa21. In addition we also sequenced the full complement of chromosomes in the Tc1 mouse and adopted a novel approach of mapping paired end sequence reads back to a reference sequence comprising mouse genome and human chromosome 21 sequence. Both approaches were a feasible way to detect structural rearrangements.

### Effect of Irradiation

In analysing the rearrangement breakpoints of the Tc1-Hsa21 at the sequence level, we observed changes consistent with non-homologous end joining rather than non-allelic homologous recombination, as might be expected from previous publications [15], [16], [17]. Unexpectedly we also found large-scale duplications, which have not been associated with DNA rearrangements as a result of radiation damage. These changes in copy number are unique to Tc1-Hsa21 and are not observed in the HT1080 cell line from which Tc1-Hsa21 was derived. The mechanism by which such large duplications could be produced is unknown [17], [18], [19] although we speculate that replication-related mechanisms may play a role. Intriguingly we found most of these duplications occurred in Giemsa dark bands suggesting that these regions are potentially either more susceptible to the effects of ionizing radiation or their duplication is better tolerated than that of gene-rich Giemsa light regions.

### Gene Expression and Copy Number in the Tc1-Hsa21 Chromosome

Despite the multiple rearrangements of Tc1-Hsa21 shown here, transcription of Hsa21 genes in Tc1 hepatocytes closely resembles that in human liver [10] and the expression of a number of other genes has also been reported in other Tc1 mouse tissues, such as brain [3]. This suggests that the genomic relocation of Hsa21 genes in the Tc1 model does not largely alter their expression; local sequence rather than the larger scale genomic context appears to be the primary determinant of gene expression.

The genes *RBM11*, *ABCC13*, *HSPA13*, *SAMSN1*, *NRIP1*, *USP25*, *c21orf31*, *S100B* and *PRMT2* are duplicated in Tc1-Hsa21 and therefore are tetrasomic in the Tc1 mouse. The expression levels of *HSPA13*, *SAMSN1*, *USP25*, *PRMT2* and *S100B* are sensitive to gene dosage [20], [21]. Thus the effect of these genes on the phenotype of the Tc1 may be important; for example in alternative mouse models, Tg3(S100b) and Tg5(S100b), the extent of astrocytosis induced by elevation of *S100B* depends on the level of expression of the gene [22].

In total, 50 genes are deleted or disrupted on Tc1-Hsa21 and so remain functionally disomic in the mouse model. Thus genes such as *RUNX1* (which may contribute to childhood leukaemia in people with DS), *APP* (a key Alzheimer disease gene) and *SYNJ1*, *RCAN1* and *ITSN1*, which have been linked to endosomal/synaptic abnormalities [23], [24], [25] are unlikely to contribute to the phenotype of this mouse model of DS.

Transgenic mice that over-express these missing genes could be crossed to the Tc1 model to investigate their influence on DS-associated phenotypes in the context of Hsa21 aneuploidy. Similarly, the genetics of the Tc1 mouse can be manipulated to restore Hsa21 genes or genomic regions of functional trisomy to disomy to investigate their role in DS [26], [27], [28].

The Tc1 mouse is trisomic for 200 Refseq genes, this compares with the Ts65Dn mouse that is trisomic for around 130 Hsa21 orthologues. The Ts65Dn model has recently been shown to be trisomic for a region of mouse chromosome 17 that is not syntenic with Hsa21 and contains around 60 genes [29], [30]. Trisomy of these genes may cause phenotypes in the model that are not related to DS. One of these studies was undertaken using NGS [29], using a similar approach to that described here. DS mouse models have also been generated by duplication of the regions of mouse chromosomes 16 (Mmu16), 10 (Mmu10) and 17 (Mmu17) that are syntenic with Hsa21 [31], [32]. The Ts1Yey mouse is trisomic for 145 genes from Mmu16, the Ts2Yey for 17 genes from mouse Mmu10 and the Ts3Yey for 60 from Mmu17. These three models can be crossed to generate a triple transgenic model that is trisomic for 222 Hsa21 orthologues [32]. However, this model is not trisomic for Hsa21 genes that do not have a mouse orthologue.

The data presented in this paper provide a ‘gold standard’ analysis of the genetics of the Tc1 mouse model, and all other mouse models derived from experiments involving XMMCT. In the case of the Tc1 mouse, the information presented here is crucial for understanding the phenotypes found in this model and how they relate to human DS, and in particular how individual genes may be involved in the syndrome. In addition this paper makes two more contributions by the novel use of massively parallel sequencing: firstly, in overcoming specific technical and bioinformatics difficulties of analysing a human chromosome on a mouse background where highly conserved sequences may confound the analysis. Furthermore, the availability of sequence data covering an entire chromosome enables for the first time an unbiased analysis of chromosome structural and DNA sequence characteristics of radiation-induced rearrangement breakpoints.

## Methods

### Ethics Statement

This study was conducted following approval by the local Ethical Review Process of the MRC National Institute for Medical Research and authorisation by the UK Home Office, Animals (Scientific Procedures) Act 1986 under relevant Project Licence authority. The ERP approved the work and reported that all work reflects contemporary best practice. High standards in the design and conduct of work have been applied in this project and full implementation and consideration of the 3Rs (where appropriate) has been made.

### Mice

The Tc(Hsa21)1TybEmcf (Tc1) mouse strain containing a copy of human chromosome 21 (Hsa21) was maintained by crossing Tc1 female mice to male B6129S8F1/Nimr mice, which were F1 progeny generated from a cross of C57BL/6J female and 129S8/SvEv male mice.

### High Resolution Microarray Analysis

Genomic DNAs extracted from a Tc1 positive mouse liver sample, a Tc1 negative mouse liver sample and the HT1080 cell line [4] were assessed for copy number imbalances on the Agilent array CGH platform. A 1 million feature microarray was designed using the Agilent eArray software (human genome build 19/NCBI37). All catalogue oligonucleotides were selected from the Agilent HD library (329772 in total). A 60mer oligonucleotide was selected approximately at every 80 basepairs using the genomic tiling feature on eArray (approximately 427125 in total). A probe group was included on the array containing an oligonucleotide every 300 kb selected across the entire human genome (approximately 6146). A pool of 100 human male individuals was used as a reference DNA. Briefly 300 ng test DNA (either Tc1+ve, Tc1-ve or HT1080) was labeled as described previously [33]. Each test DNA was combined with a labeled reference DNA sample and hybridised to a microarray following the Agilent Oligonucleotide Array-Based CGH for Genomic DNA Analysis procedure G4410-90010 (version 6.2). Post washing the microarrays were scanned using an upgraded Agilent scanner at 3 microns and 20 bit images were generated. The Cy3 and Cy5 intensity values for each feature were extracted from the microarray array scanned image using Feature Extraction software version 10.5.1.1.

The two fluorescence intensity distributions were then adjusted independently towards their geometric mean using the R package “aCGH.Spline”. This method used a cubic spline interpolation and outlier extrapolation to account for non-standard dye biases (the experimental design results in a large dye bias and number of outliers). A custom wavelet method was used to remove the presence of genomic waves and the Tc1-ve littermate log2 ratio values were subtracted from the Tc1+ve data to produce the final log2 ratio profile (Fig. 1).

### Copy Number Detection in High-resolution Microarray Data

The calling algorithm GADA was run on the microarrayCGH log2 ratio data after having removed all microarray data points where the oligonucleotide had a quality score of >0.6. Oligonucleotide probe scores are an indication of the uniqueness of a probe sequence. Probes with a score of 1 are unique and highly reliable in performance on a microarray. Probes with a low score are more likely to have a repetitive sequence and will consequently not report as quantitatively. Probe scores can be generated using eArray (https://earray.chem.agilent.com/earray/). Calls were made using the criteria of 10 probes or more needed to report a call and an absolute log2 ratio of 0.5 (Tables S1, S2).

### Genomic DNA Isolation

Genomic DNA was prepared from Tc1 positive and negative mouse tail or liver samples using a Qiagen midi kit. Briefly 80 mg mouse tail or liver tissue was thoroughly homogenised mechanically using an IKA T10 homogeniser in G2 buffer in the presence of RNase A according to the manufacturer’s protocol. The homogenate was treated with Qiagen Protease in a 2 hour incubation at 50°C. Genomic DNA was then purified by passing through a Qiagen Genomic-tip following the manufacturer’s protocol. Eluted genomic DNA was precipitated in 0.7 volumes of isopropanol, spooled into TE buffer, pH 8.0, and dissolved overnight at 50°C.

### Stimulation of Mouse Spleen Cells

B lymphocytes from Tc1 positive mouse spleens were prepared and stimulated using lipopolysaccharide (LPS, Sigma) as described previously [34]. After 44–48 hours of culture in LPS (50 µg/ml), the stimulated culture was blocked in metaphase with 0.1 µg/ml demecolcine (Sigma) for 3.5 hours prior to harvesting.

### Chromosome Preparation and Sorting

Chromosomes were prepared as described previously [35] and stained overnight with Hoechst (HO, Sigma) and chromomycin A3 (CA3, Sigma). The stained chromosomes were treated overnight to a final concentration of 25 mM sodium sulphite (Sigma) and 10 mM sodium citrate (Sigma) before flow analysis and sorting.

Stained chromosome suspensions were analyzed on a flow cytometer (Mo-Flo®, Beckman Coulter) equipped with two water-cooled lasers (Coherent, Innova 300 series) as described previously [35]. The fluorescence of HO, CA3, forward scatter (FSC) and pulse width parameters were collected. A gated region was set on the plot of linear FSC versus linear pulse width to exclude clumps and debris (Fig. S1a), and the bivariate plot of HO versus CA3 fluorescence was gated on this region (Fig. S1b). A total of 100,000 events were acquired and analyzed using Summit analysis software. This is the first published flow karyogram for the Tc1 mouse (Fig. S1b).

The stained chromosome suspension was flow sorted at a data rate of 8,000–10,000 events per sec as described previously [36]. 250,000 copies of chromosome 21 were flow sorted in chromosome sheath buffer (10 mM Tris-HCl (pH8.0), 1 mM EDTA, 100 mM NaCl, 0.5 mM sodium azide) into a sterile UV treated 1.5 ml eppendorf tubes with a final volume of 250 µl.

DNA was extracted by incubation with 6/100 volume 0.25 M EDTA/10% sodium lauroyl sarcosine and 1/100 volume proteinase K (20 mg/ml) overnight at 42°C. This was followed by incubation with 1/100 volume phenylmethylsulphonyl fluoride (PMSF, 4 mg/ml) for 40 minutes at room temperature. The DNA was then precipitated with the addition of 4/100 volume of NaCl (5 M), 8/1000 volume of pellet paint (Novagen) and 3 volume of 100% ethanol.

### Next Generation Paired-end Sequencing of Tc1-Hsa21

A total of five Illumina paired-end small and large insert libraries were prepared as detailed in Table S3. For paired-end high complexity libraries the manufacturer’s protocol was followed. The paired-end no PCR library was prepared according to Kozarewa *et al* 2009 [37]. A large insert paired-end library which was double size selected was prepared according to Quail *et al*., 2008 [38]. Prepared libraries were sequenced using standard Illumina protocols (GenomeAnalyzer, Illumina).

### Sequence Read Mapping and Analysis

Mapping and identification of structural rearrangement events was performed by the paired read pipeline implemented by the Cancer Genome Project at the Sanger Institute as described in Campbell *et al*., [39]. Paired-end reads were mapped to a composite reference of the human reference sequence for chromosome 21 (GRCh37) and the mouse genome reference (NCBI m37) using MAQ 0.7.1–6 (developed by Heng Li, http://www.sanger.ac.uk/Users/lh3/). Reads in which the two ends failed to align to the genome in the correct orientation and distance apart were further screened with the SSAHA algorithm24.

Estimation of insert size and insert size thresholds was performed on the first 5000 correctly orientated paired-end sequence reads with an insert size greater than 0, from the first lane of sequencing from each library. Paired-read insert sizes were binned at 10 base pair increments and plotted as histograms. The top of the curve indicated the most frequent library insert size and the upper insert size threshold was found by looking for a ratio of less than 1.1 between 10 base pair increments. In addition a safety margin of 50 bp is added to upper insert size threshold. Following mapping, the reads are processed to remove duplicate pairs. Duplicate removal was achieved by removing reads that have exactly the same mapping coordinates. Reads were assigned a quality score and reads with scores greater than 35 were used to describe structural rearrangements.

### Copy Number Determination from Mapped Sequence Read Pairs

Reads with flags of 18, (short correct pairs) 20 (long correct) or 130 (paired correct by smith waterman) were binned by leftmost coordinate at 1 kb intervals. Plotting frequency of reads from all library data in 1 kb bins against chromosome position produces a copy number plot (Fig. 1).

### Structural Rearrangement (SR) Analysis

SR events are identified by processing all read pairs with quality scores >35 where read pairs map at distances greater than the insert size thresholds of the library. An SR event is then identified by clustering paired-reads that describe the same high-quality SR event. The normal orientation of the two paired-read sequences is forward (+) and reverse (−) i.e. the first sequence maps to the primary strand whilst the second sequence maps to the complimentary strand. Variation in this signature describes a structural variation. The four possible scenarios are:

- Forward Reverse (+−), normal mapping signature; however +− indicates deletion if the two coordinates indicate a distance larger than the library insert size.
- Forward Forward (++), second read indicates inverted orientation.
- Reverse Forward (−+), tandem duplication.
- Reverse Reverse (–), first read indicates inverted orientation.

Rearrangement events in the Tc1 Hsa21 were compiled from the clustered file. Rearrangement events were predominantly identified from clusters with a read count of two or more and detected in 3 or more libraries (Table 1). These were then confirmed by local assembly using reads within 4 kb of the breakpoints of the rearrangement event and by PCR verification.

The proportion of aberrantly mapping read pairs is vastly biased to the short arm of chromosome 21 sequence. 1022 of 1471 aberrantly mapping read pairs have the first chromosome position mapping to sequence on the short arm of chromosome 21. Five aberrantly mapping read pairs (Table 1) were only present in large insert library data sets suggesting a combination of large and small paired-end sequencing libraries is an effective strategy to capture maximum rearrangement data.

To determine the structure of Tc1 Hsa21, a breakpoint at copy number 1 requires an aberrant read pair linking each side of the breakpoint to permit the repositioning of sequence proximal and distal to the breakpoint. However a breakpoint at a copy number change point may only have one aberrant read pair repositioning the sequence at a copy number of 2 and the wild type sequence may remain intact.

### Fluorescence *in situ* Hybridisation Analysis of Tc1-Hsa21

Metaphase chromosomes were prepared from a lipopolysaccharides (LPS)-stimulated spleen culture from Tc1 positive mice following the published protocols [34]. A centromeric probe (plasmid, p1Z2A) and a commercially available telomeric probe (s21qter, KBI-40238, Kreatech) were hybridised to metaphase chromosomes prepared from Tc1+ve metaphase chromosomes following a standard metaphase FISH protocol detailed below, whilst a pan-telomeric probe (Telomeric PNA probe, K5326, DAKO) was hybridised using the manufacturer’s suggested protocol. Images were captured on Zeiss Axioplan 2 imaging fluorescence microscope using SmartCapture software (Digital Scientific UK).

Bacterial artificial chromosome (BAC) clones were selected from human chromosome 21 sequence at approximately 2 Mb intervals and fosmid clones were selected in regions of the six Tc1 Hsa21 duplications (Table S5). DNA was purified using a PhasePrep BAC DNA kit (Sigma) following manufacturer’s protocol and amplified using a whole genome amplification kit (WGA2, Sigma) following manufacturer’s protocol. The WGA2 products were then labeled using a whole genome amplification kit (WGA3, Sigma) with Biotin-16-dUTP (Roche), ChromaTide™ Texas Red®-12-dUTP (Invitrogen), Green-dUTP (Abbott), Cy3-dUTP and Cy5-dUTP (Enzo). A master mix was made using 16.7 µl PCR water, 2.5 µl 10× Amplification master mix (A5606, Sigma), 2.5 µl dNTP mix (2 mM dATP, 2 mM dCTP, 2 mM dGTP, 1.4 mM dTTP, Abgene), 0.5 µl 50 mM MgCl_2_ (Bioline), 1.5 µl dUTP (biotin-, green-, cy3- or cy5-dUTP) and 0.3 µl BioTAQ (Bioline) per reaction. This was added to 1 µl WGA DNA to make a total reaction volume of 25 µl and cycled once for 3 minutes at 95°C, followed by 18 cycles of 94°C for 15 seconds and 65°C for 5 minutes. The same PCR cycles were used for ChromaTide™ Texas Red®-12-dUTP labelling with the following master mix; 17.7 µl PCR water, 2.5 µl 10× Amplification master mix (A5606), 2.5 µl dNTP mix (2 mM dATP, 2 mM dCTP, 2 mM dGTP, 1.8 mM dTTP, Abgene), 0.5 µl 50 mM MgCl_2_, 0.5 µl of ChromaTide™ Texas Red®-12-dUTP and 0.3 µl BioTAQ per reaction. Probes were cut using 1 µl 10 µg/ml DNase 1 (0.4 ug/ml number of DNase 1 units required may vary per experiment) for 90 min at 15°C and then checked on a 1% agarose gel. If the sizes of DNA fragments were around 300 bp the reaction was stopped by adding 1 µl 0.5 M EDTA per tube (20 mM) and then heating to 65°C for 10 minutes.

Labeled BAC clones combined with Human Cot-1 DNA (Invitrogen) were denatured in hybridisation buffer (50% formamide (ACROS), 2×SSC, 10% dextran sulphate, 0.5 M phosphate buffer, pH 7.4) at 65°C and hybridised onto dry metaphase slides that had been treated with pepsin, aged on a 65°C hot plate for an hour, denatured in 70% formamide/2×SSC for 1.5 minutes, and dehydrated in an ethanol series. Slides were hybridised at 37°C overnight and post-hybridisation washes (two 50% formamide/2×SSC washes then two 2×SSC washes, all for 5 minutes at 43°C) were carried out. When probes were labeled indirectly (biotin) the slides were detected using Cy5.5 anti-biotin antibody (5 µg/ml, Tebu-bio Ltd) and then washed in 4×SSC with 0.05% Tween 20. Slides were mounted using an antifade mounting solution with DAPI (Slowfade Gold with DAPI, Invitrogen) and sealed with nail varnish.

Assembly of Tc1-Hsa21 regions utilising aberrantly mapping read pair data organised Tc1-Hsa21 into unjoined sections (Table S5). Sequential hybridisation of BAC and fosmid clones (Fig. S2, Table S6) aided the positioning and orientation of the majority of the larger sections within the structure of Tc1-Hsa21 (Table S5, Fig. 3). Acrocentric regions remain unpositioned within the Tc1-Has21 structure.

All six duplications (identified by fosmids fd1-fd6) gave two signals; one signal on each Tc1 Hsa21 arm (Fig. S2 k-p). Duplications 1–3 and one copy of the duplication 4 were placed within chromosomal regions by sequence data, whilst the precise position of the other copy duplication 4 and duplications 5 and 6 are undeterminable by FISH due to the resolution of the technique and the size of the target chromosome.

### Verification of Structural Rearrangements by PCR and Capillary Sequencing

PCR primers were designed to amplify predicted breakpoint junction fragments. Primer details are listed in Table S7 (primers were designed using human genome build 19). PCR amplifications were performed on Tc1 positive mouse genomic DNA, Tc1 negative mouse genomic DNA, genomic DNA from 35 different female individuals (UK blood donors, University of Cambridge & NHS Blood and Transplant Cambridge), and genomic DNA from the HT1080 cell line (Fig. S4). For PCR products less than 1 kb the PCR conditions were as follows: 100/200 ng genomic DNA was amplified in a 15 µl reaction containing 400 µM each dNTP, 1.3 µM each primer and 0.45 units Thermo Start *Taq* DNA polymerase (ABgene), in 1× PCR buffer1 (Thermo Scientific). PCR was performed with an initial denaturation step at 95°C for 15 minutes followed by 30 cycles of: 95°C for 30 seconds, 60°C for 30 seconds, 72°C for 30 seconds and a final extension step at 72°C for 10 minutes. For PCR products larger than 1 kb the PCR conditions were as follows: 100/200 ng genomic DNA was amplified in a 25 µl reaction containing, 300 µM each dNTP, 0.8 µM each primer, 2.5 units HotStarTaq DNA polymerase (Qiagen) and 0.2 units HotStar HighFidelity DNA polymerase (Qiagen). PCR was performed with an initial denaturation step at 95°C for 15 minutes followed by 40 cycles of: 95°C for 20 seconds, 57°C for 1 minute, 68°C for 10 minutes and a final extension step at 68°C for 10 minutes PCR products were visualised on 2.5/0.6% gels and results are shown in (Fig. S4). The breakpoints listed in Table 1 were all confirmed to be unique rearrangements in Tc1 positive mice and not present in the original HT1080 genomic DNA or in 35 normal individuals. All junction fragments were capillary sequenced as follows: Cleaned fragments were sequenced from both ends with the appropriate primers (Table S7) using di-deoxy chain termination method, with V.3.1 Big Dye Terminator Chemistry. The resulting sequencing reactions were analysed on a 3700 ABI sequencing machine. Breakpoint sequences were blasted back to NCBI37 and aligned to reference genome sequence using ClustalW2 (http://www.ebi.ac.uk/Tools/clustalw2). This allowed breakpoint mapping to the base pair level (Fig. S3).

### Confirmation of Potential Fusion Gene Transcription:RNA Extraction and RT-PCR

RNA was extracted from whole brains from 6- to 10-week old Tc1 positive mice and age and sex matched non-transchromosomic controls. Total RNA was extracted using TRIzol reagent (Invitrogen), precipitated as per manufacturer’s instructions and resuspended in DNase-free water. Human brain total RNA was supplied by Ambion. Amounts of RNA were equalised and cDNA was generated using a standard reverse-transcription protocol using random primers (Promega), Superscript II (Invitrogen), First Strand Buffer (Invitrogen) and dNTPs (Promega). PCR using primers designed to NDUFV3 (ENSE00001436763 NDUFV3f1 5′-TGTTTGCTGCGGCAAGGAC-3′ NDUFV3f2 5′- AGCTGCTGTGGCCCTGCTTG-3′) and PCBP3 (ENSE00001682409 PCBP3R1 5′-CTCCCTGATCTCCTTGATCTTG-3′ and ENSE00001303536 PCBP3R1 5′- TCCAGCATGACCACACAGATCTG-3′) were used to check expression of the novel fusion gene (Fig. S5).

### SNP Calling

Paired-end reads were mapped to a composite reference of the human reference sequence for chromosome 21 (GRCh37) and the mouse genome reference (NCBI m37) using BWA version 0.5.7 (1). Duplicate fragments were marked per library using picard and qualities recalibrated using GATK. SNPs were called using samtoolsSnp. SNP consequences were assigned based on gencode annotation (in Ensembl version 57). SNPs were assigned a dbSNP reference where available. Homozygous SNPs include a splice site mutation in the pseudogene, CR392039 and four non-synonymous Tc1-Hsa21 coding mutations. An amino acid substitution, at a position conserved in mouse and rat, was detected in the steroid co-repressor, *NRIP1* that is up-regulated in DS [40]. Non-synonymous changes also occur at conserved sites in the keratin associated protein family genes KRTAP10-10 and *KRTAP6-1* that function in hair development [41], [42]. A mutation in the final coding exon of the testis-expressed gene *UMODL1* results in the substitution of a conserved alanine [43].

## Supporting Information

Figure S1 Flow sorting of Tc1-Hsa21, a) a plot of linear forward scatter (FSC) versus linear pulse width showing a gated region set to exclude debris; b) bivariate plot of HO versus CA3 fluorescence with Tc1-Hsa21 peak circled in red.
(TIF)

Figure S2 Tc1-Hsa21 metaphase chromosomes hybridised with fluorescently labelled clones, a–j) BAC 2,3,5,6,8,10,11,15,16,17,19 refers to BAC I.D. number (Table S5, S6), fs1 is a fosmid (Table S5, S6), fT is a fosmid which contains the TPTE gene sequence, CX is a fosmid which contains the CXADR gene sequence, white c is centromere, k–p) white signal is centromere, green hybridisation signal is BAC 8, red signal is (k) fosmid fd1 (l) fosmid fd2, (m) fosmid fd3, (n) fosmid fd4, (o) fosmid fd5, (p) fosmid fd6, respectively, note duplication signals on each arm. Below schematic shows the relative position of FISH probes on Tc1-Hsa21. Duplications are coloured green. Duplicated regions that cannot be ordered by FISH are boxed above, c is centromere, size of chromosomal region is in megabases.
(TIF)

Figure S3 Breakpoint junction fragment sequence aligned to human chromosome 21 reference sequence, sequence originating from forward strand (→) or reverse strand **(**←**)** as indicated**.** Figure shows the exact sequence of the junction fragments observed at the breakpoints, **a)** 10603042 (←) and 47916774 (→), **b)** 10739714 (←) and 26376257 (←), **c)** 11167165 (→)and 25728012 (←), **d)** 15970237(→) and 16083578 (→), **e)** 17769030 (←) and 26038660 (→), **f)** 17764356 (→) and 17771310(←), **g)** 17770361 (←) and 19761690(→), **h)** 18734159(→) and 24880958(→), **i)** 20514026(→) and 24460819(←), **j)** 20514023(←) and 20756929(→), **k)** 20756922(→) and 23648470(→), **l)** 20893994 (→) and 23648476(←), **m)** 20893996(←) and 24725875(←), **n)** 22612235(→) and 23294101(←), **o)** 23198373(←) and 23306917(→), **p)** 23306930(→) and 25728022(→), **q)** 26038424(→) and 27263316(←), r) 33223436 (→) and 36370289(→), **s)** 33223573(←) and 45795618(→), **t)** 37017239(←) and 45795619(←), **u)** 44314438(→) and 47322101(→), **v)** 46868313 (→) and 47916772(←). Transition from blue to red text marks the precise breakpoint position. Bases in boxed purple could originate from either reference sequence. Underlined bases are inserted at the breakpoint.//indicates additional base pairs inserted, see Table 1 for details.
(TIF)

Figure S4 PCR verification of structural rearrangements, breakpoint junction fragments were amplified by PCR. These were found to be unique to Tc1-Hsa21 and were not found in 35 human control genomes. For example, primers specific for ←10603042 and 47916774 → were used to raise a 1500 base pair product across this breakpoint that is only observed in Tc1 genomic DNA. PCR products were separated on a 2.5% Agarose gel. Tc1+, genomic DNA from a Tc1 positive mouse, Tc1 −, genomic DNA from a Tc1 negative mouse, HT1080, genomic DNA from HT10 cell line, 1–35, genomic DNA samples from 35 different individuals.
(TIF)

Figure S5 RT-PCR verification of fusion gene transcription, a rearrangement of Hsa21 in the Tc1 mouse (chr21∶46868268+47916724+) was predicted to form a fusion of gene consisting of the first exon of *NDUFV3* and the final 9 exons of *PCBP3.* The expression of this novel transcript was verified by RT-PCR of whole brain RNA isolated from Tc1 and control mice and a human brain RNA sample supplied by Ambion (*NDUFV3f1* 5′-TGTTTGCTGCGGCAAGGAC-3′ *PCBP3r1* 5′-CTCCCTGATCTCCTTGATCTTG-3′ predicted size 177 base pairs).
(TIF)

Table S1 Copy number changes detected in Tc1 Hsa21 by high resolution aCGH. Details of the hybridisation of Tc1 genomic DNA to the CGH array, delineating contiguous regions (start, stop) that have a similar log2 hybridisation ratio, (base pair positions according to human genome build 19), thus identifying the break-points of copy number changes on the chromosome. The number of known copy number variations (CNVs) in each region (Conrad et al., 2010, Nature, **464**, 704-12) and the % overlap between the regions found in Tc1-Hsa21 and the known CNV.
(XLS)

Table S2 Copy number changes detected in HT1080 cell line DNA by high resolution aCGH. Details of the hybridisation of HT1080 genomic DNA to the CGH array, delineating contiguous regions (start, stop) that have a similar log2 hybridisation ratio, (base pair positions according to human genome build 19), thus identifying the break-points of copy number changes on the chromosome. The number of known copy number variations (CNVs) in each region (Conrad et al., 2010, Nature, **464**, 704-12) and the % overlap between the regions found in HT1080 and the known CNV.
(XLS)

Table S3 Details of NGS libraries prepared from Tc1 mice and obtained sequence yields, number of correctly mapping reads and achieved sequence fold coverage on chromosome 21.
(XLS)

Table S4 RefSeq genes (The NCBI RefSeqGene Project, http://www.ncbi.nlm.nih.gov/projects/RefSeq/RSG/, RefSeq release no.42) on human chromosome 21. The copy number and rearrangement status of each gene is listed.
(XLS)

Table S5 41 regions of Tc1 Hsa21 delineated by a rearrangement breakpoint or a copy number change point, arranged by NGS rearrangement data and ordered by FISH mapping data. The orientation of each region of Tc1 Hsa21 is indicated relative to genomic Hsa21 forward (F) or reverse (R) strand. The order of regions 11, 20, 22, 24, 19, 18, 25, 17, 13, 28, 26, 6, 15, 35, 32, 5, 9, 10, 33, 38, 40, 36, 37, 31, 29, 23, 23, 22, 21, 20, and 11 in this table are based on FISH mapping data (Fig. S2). The positions of the smaller acrocentric regions 1, 2, 3, 7 and 8 were not placed by FISH and are placed in this table arbitrarily. The FISH data suggests that the smaller duplicated regions 26, 30 and 41 have a copy on each arm of Tc1-Hsa21, their position in this Table is suggested by FISH within the resolution of the technique but not precisely known. *Region 12 is triplicated but the position of the other two copies is unknown. The position of region 27 (24725876–24880958) remains unknown (no gene content). fT, FISH probe for TPTE gene, CX, FISH probe for CXADR gene, fs1 fosmid, fd1-fd6 are fosmids for 6 duplications (for details see Table S6). The data in this table were used to draw Figure 3. Darker borders delineate regions joined by aberrant read-pair sequence data. Regions 14, 16, 34 and 39 are deleted.
(XLS)

Table S6 Details of the bacterial artificial clones and fosmid clones used as *in situ* hybridisation probes.
(XLS)

Table S7 Rearrangement breakpoints unique to Tc1 Hsa21 and their consequence to gene disruption. Breakpoint position at base pair resolution obtained by sequencing a PCR amplified junction fragment (column 1); ▪, indicates breakpoint was not PCR verified so position is not accurate to base pair resolution; •, indicates the rearrangement was only detected in paired sequence read data obtained in large insert libraries; †† indicates breakpoint is between 24880910 and 24880958, * indicates insertion that’s only known homology is with a region on chromosome 4 (chr4_gl000194_random, 190017–190455); Un, indicates bases of unknown origin; **, indicates chr21 9826532–9826580; ▴ indicates bases 23648470–23648476 appear in 2 breakpoint junction fragments, † indicates bases 23306917–23306930 appear in 2 breakpoint junction fragments; ♦, indicates AT repeat at the breakpoint is most likely to come from the 44314438 side of the breakpoint. Underlined bases are inserted at the breakpoint. Bold bases could originate from either reference sequence. The rearrangement between c21orf34 and TMPRSS15 is part of a very complex rearrangement around 17.7 Mb (hg19), which remains unresolved. Primer sequences and positions used to amplify junction fragments (columns 2–7). Genes disrupted at the breakpoints (columns 8–9).
(XLSX)

Table S8 Stop gained, splice site, non synonymous and synonymous SNPs detected NGS reads obtained from 5 paired end libraries unique to Tc1 Hsa21 and not reported in db SNP. Base pair coordinate relative to SNP NCBI37.
(XLS)
