# Supplementary figures and images for: Massively Parallel Sequencing Reveals the Complex Structure of an Irradiated Human Chromosome on a Mouse Background in the Tc1 Model of Down Syndrome
Source: PLoS One. 2013 Apr 15;8(4):e60482. doi: 10.1371/journal.pone.0060482 (PMC3626651; doi:10.1371/journal.pone.0060482)

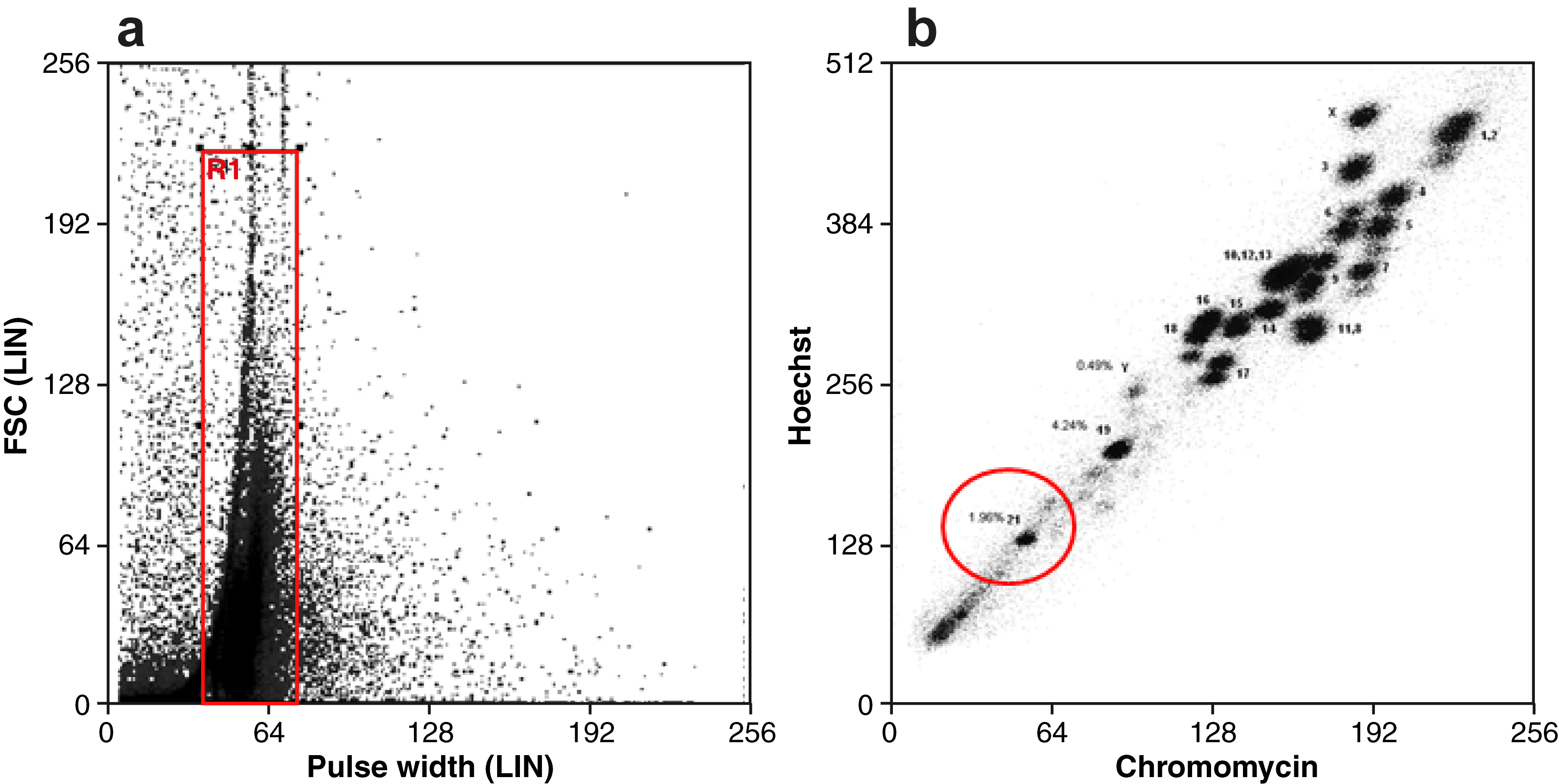

Supplement: Figure S1 — Flow sorting of Tc1-Hsa21, a) a plot of linear forward scatter (FSC) versus linear pulse width showing a gated region set to exclude debris; b) bivariate plot of HO versus CA3 fluorescence with Tc1-Hsa21 peak circled in red. (TIF) [file pone.0060482.s001.tif]

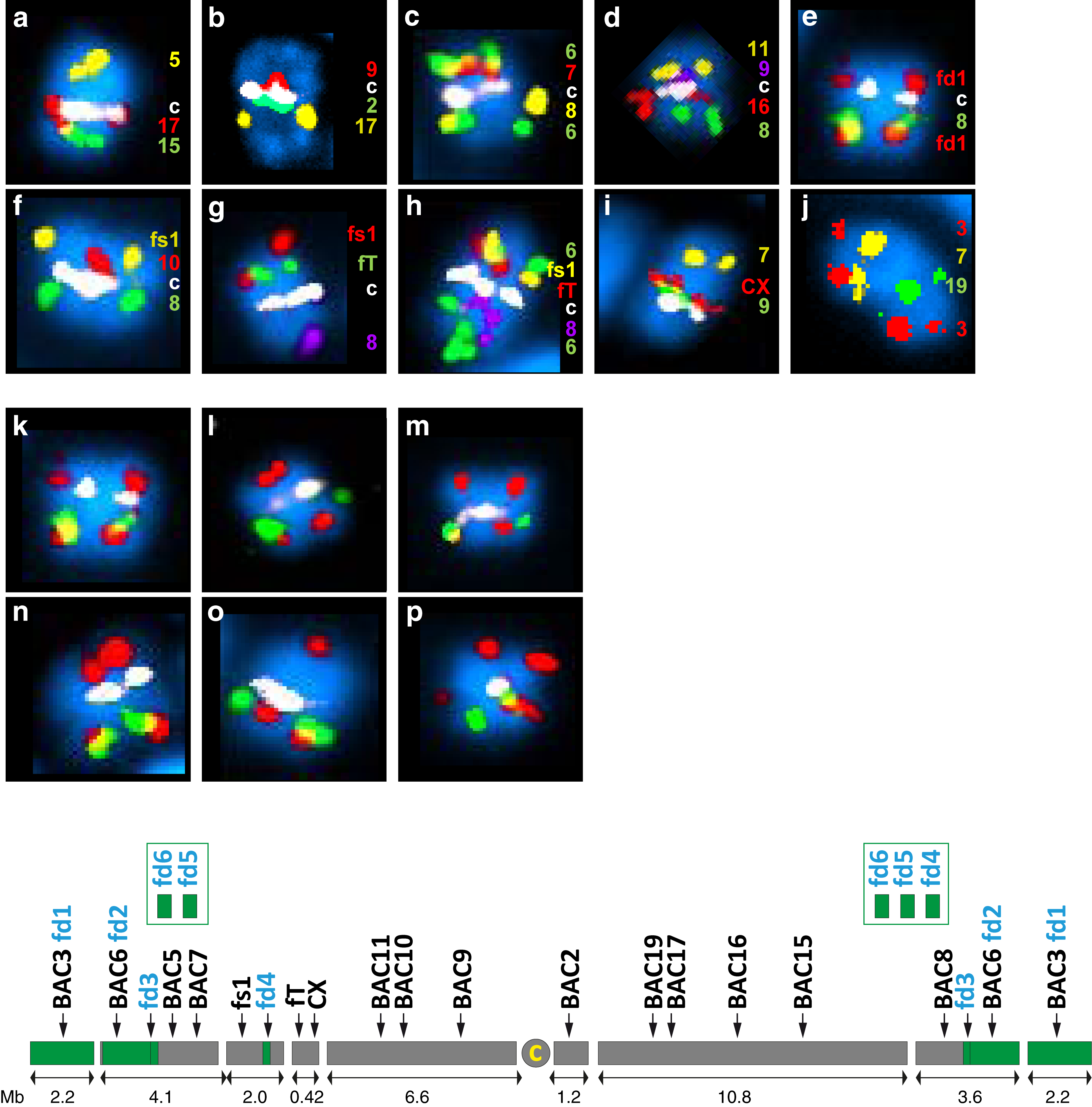

Supplement: Figure S2 — Tc1-Hsa21 metaphase chromosomes hybridised with fluorescently labelled clones, a–j) BAC 2,3,5,6,8,10,11,15,16,17,19 refers to BAC I.D. number (Table S5, S6), fs1 is a fosmid (Table S5, S6), fT is a fosmid which contains the TPTE gene sequence, CX is a fosmid which contains the CXADR gene sequence, white c is centromere, k–p) white signal is centromere, green hybridisation signal is BAC 8, red signal is (k) fosmid fd1 (l) fosmid fd2, (m) fosmid fd3, (n) fosmid fd4, (o) fosmid fd5, (p) fosmid fd6, respectively, note duplication signals on each arm. Below schematic shows the relative position of FISH probes on Tc1-Hsa21. Duplications are coloured green. Duplicated regions that cannot be ordered by FISH are boxed above, c is centromere, size of chromosomal region is in megabases. (TIF) [file pone.0060482.s002.tif]

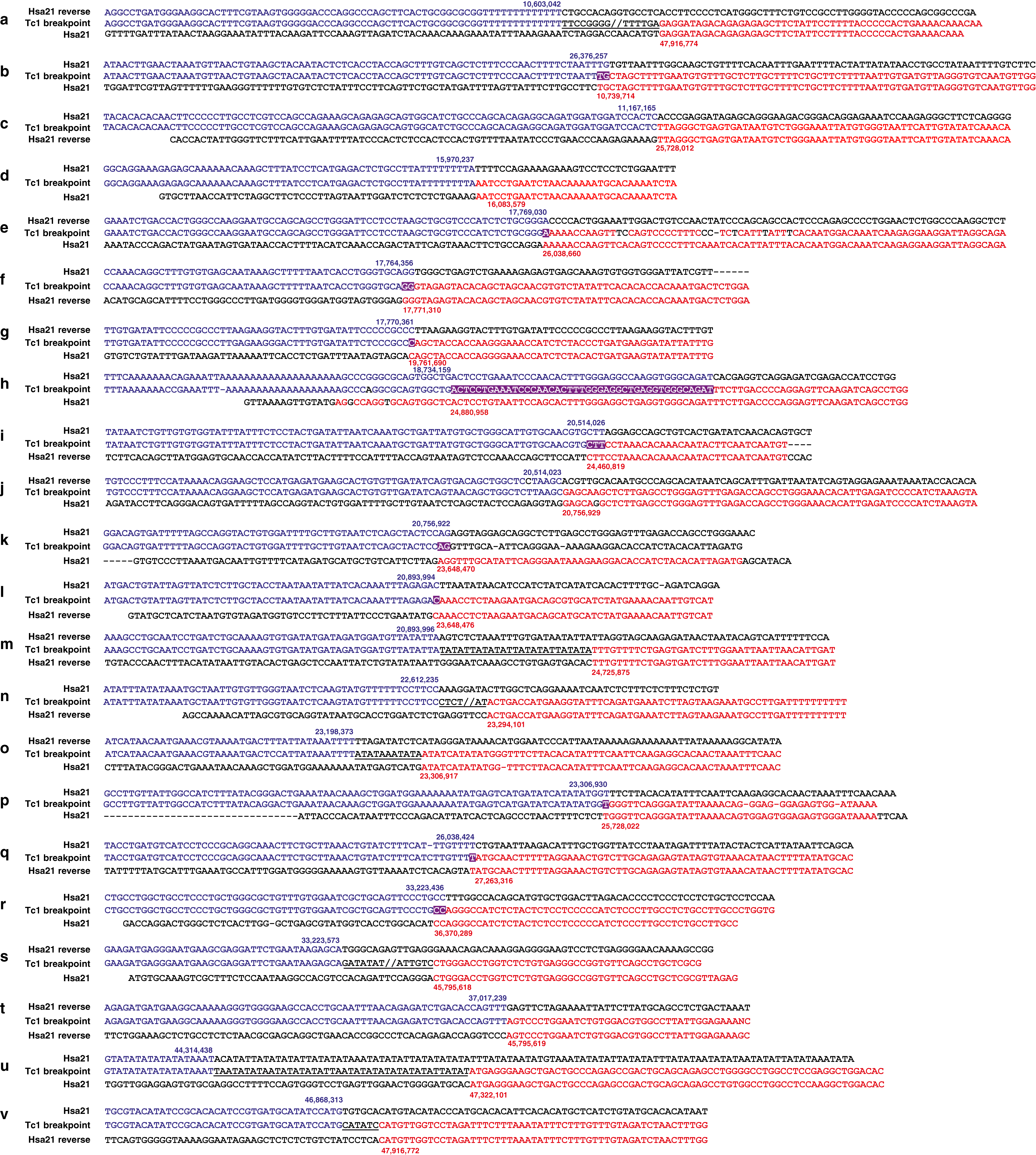

Supplement: Figure S3 — Breakpoint junction fragment sequence aligned to human chromosome 21 reference sequence, sequence originating from forward strand (→) or reverse strand (←) as indicated. Figure shows the exact sequence of the junction fragments observed at the breakpoints, a) 10603042 (←) and 47916774 (→), b) 10739714 (←) and 26376257 (←), c) 11167165 (→)and 25728012 (←), d) 15970237(→) and 16083578 (→), e) 17769030 (←) and 26038660 (→), f) 17764356 (→) and 17771310(←), g) 17770361 (←) and 19761690(→), h) 18734159(→) and 24880958(→), i) 20514026(→) and 24460819(←), j) 20514023(←) and 20756929(→), k) 20756922(→) and 23648470(→), l) 20893994 (→) and 23648476(←), m) 20893996(←) and 24725875(←), n) 22612235(→) and 23294101(←), o) 23198373(←) and 23306917(→), p) 23306930(→) and 25728022(→), q) 26038424(→) and 27263316(←), r) 33223436 (→) and 36370289(→), s) 33223573(←) and 45795618(→), t) 37017239(←) and 45795619(←), u) 44314438(→) and 47322101(→), v) 46868313 (→) and 47916772(←). Transition from blue to red text marks the precise breakpoint position. Bases in boxed purple could originate from either reference sequence. Underlined bases are inserted at the breakpoint.//indicates additional base pairs inserted, see Table 1 for details. (TIF) [file pone.0060482.s003.tif]

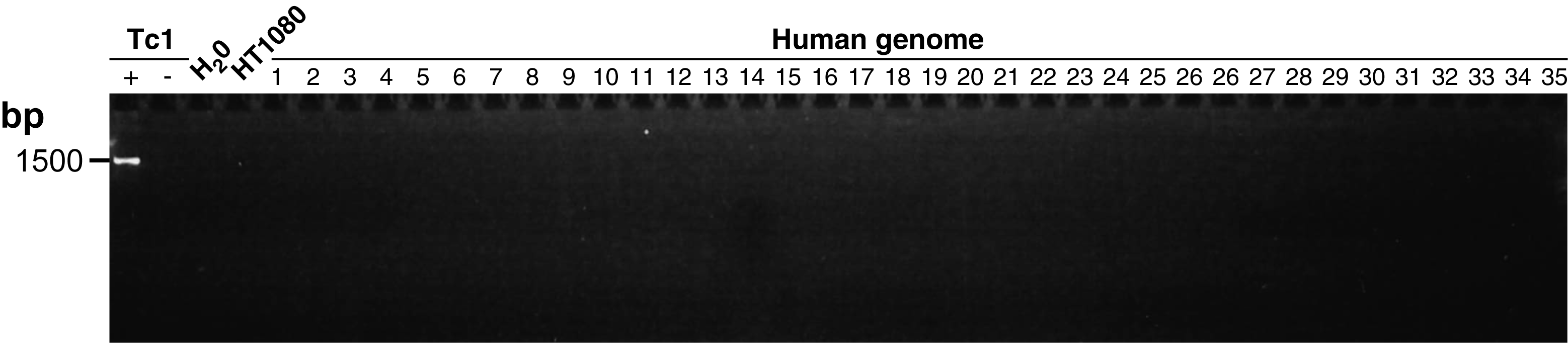

Supplement: Figure S4 — PCR verification of structural rearrangements, breakpoint junction fragments were amplified by PCR. These were found to be unique to Tc1-Hsa21 and were not found in 35 human control genomes. For example, primers specific for ←10603042 and 47916774 → were used to raise a 1500 base pair product across this breakpoint that is only observed in Tc1 genomic DNA. PCR products were separated on a 2.5% Agarose gel. Tc1+, genomic DNA from a Tc1 positive mouse, Tc1 −, genomic DNA from a Tc1 negative mouse, HT1080, genomic DNA from HT10 cell line, 1–35, genomic DNA samples from 35 different individuals. (TIF) [file pone.0060482.s004.tif]

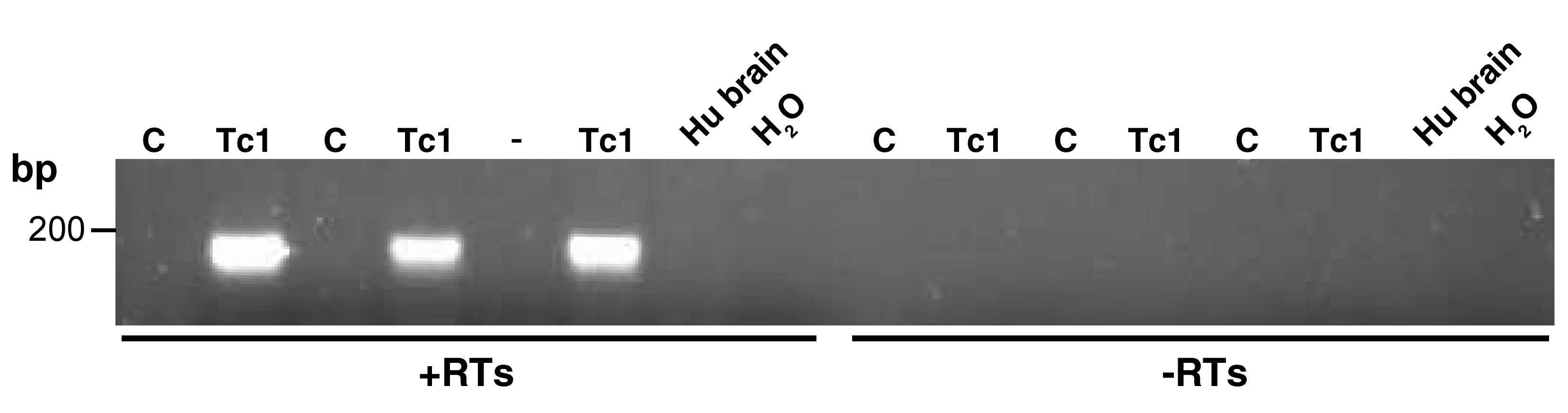

Supplement: Figure S5 — RT-PCR verification of fusion gene transcription, a rearrangement of Hsa21 in the Tc1 mouse (chr21∶46868268+47916724+) was predicted to form a fusion of gene consisting of the first exon of NDUFV3 and the final 9 exons of PCBP3. The expression of this novel transcript was verified by RT-PCR of whole brain RNA isolated from Tc1 and control mice and a human brain RNA sample supplied by Ambion (NDUFV3f1 5′-TGTTTGCTGCGGCAAGGAC-3′ PCBP3r1 5′-CTCCCTGATCTCCTTGATCTTG-3′ predicted size 177 base pairs). (TIF) [file pone.0060482.s005.tif]
